# Intraoperative Techniques That Define the Mucosal Margins of Oral Cancer In-Vivo: A Systematic Review

**DOI:** 10.3390/cancers16061148

**Published:** 2024-03-14

**Authors:** Klijs J. de Koning, Carleen M. E. M. Adriaansens, Rob Noorlag, Remco de Bree, Robert J. J. van Es

**Affiliations:** Department of Head and Neck Surgical Oncology, University Medical Center Utrecht, Heidelberglaan 100, 3584 CX Utrecht, The Netherlands

**Keywords:** oral squamous cell carcinoma, systematic review, mucosal margin, diagnostic accuracy autofluorescence, iodine, narrow band imaging

## Abstract

**Simple Summary:**

This systematic review evaluates techniques defining adequate mucosal margins during the resection of oral squamous cell carcinoma (SCC). Residual SCC and dysplasia demand distinct adjuvant treatment, such as re-resection and radiation for SCC or CO_2_-laser evaporation for severe dysplasia, necessitating accurate differentiation between SCC and dysplasia during surgery. The study includes eight investigations into margin visualization techniques—autofluorescence, iodine staining, and narrow-band imaging—concluding that, except for autofluorescence, there is considerable variability in negative predictive values. Autofluorescence does not significantly enhance margin outcomes compared to conventional white light-guided surgery, while iodine does. Studies on narrow-band imaging did not report a comparison with a white light-guided surgery cohort. The review advocates for more comprehensive studies comparing the diagnostic accuracy of iodine staining or narrow-band imaging, with a specific focus on diagnostic accuracy and the discrimination between SCC and dysplasia.

**Abstract:**

Background: This systematic review investigates techniques for determining adequate mucosal margins during the resection of oral squamous cell carcinoma (SCC). The primary treatment involves surgical removal with ≥5 mm margins, highlighting the importance of accurate differentiation between SCC and dysplasia during surgery. Methods: A comprehensive Embase and PubMed literature search was performed. Studies underwent quality assessment using QUADAS-2. Results: After the full-text screening and exclusion of studies exhibiting high bias, eight studies were included, focusing on three margin visualization techniques: autofluorescence, iodine staining, and narrow-band imaging (NBI). Negative predictive value (NPV) was calculable across the studies, though reference standards varied. Results indicated NPVs for autofluorescence, iodine, and NBI ranging from 61% to 100%, 92% to 99%, and 86% to 100%, respectively. Autofluorescence did not significantly enhance margins compared to white light-guided surgery, while iodine staining demonstrated improvement for mild or moderate dysplasia. NBI lacked comparison with a white light-guided surgery cohort. Conclusions: We recommend studying and comparing the diagnostic accuracy of iodine staining and NBI in larger cohorts of patients with oral SCC, focusing on discriminating between SCC and (severe) dysplasia. Furthermore, we advise reporting the diagnostic accuracy alongside the treatment effects to improve the assessment of these techniques.

## 1. Introduction

Approximately one-third of all head and neck cancers are oral squamous cell carcinoma (SCC) [1]. The preferred choice of treatment is complete surgical removal with histopathological adequate resection margins of the primary tumor to establish local control [2,3].

There is still a discussion about the definition of an adequate margin. Several studies investigated the ideal histopathological cutoff margin [3,4,5,6,7,8]. Most guidelines define a free margin as ≥5 mm between the SCC and the resection plane [9,10]. There is a general consensus that margins between 0 and 1 mm from the resection plane adversely affect locoregional survival [7,11,12] and are an indication for adjuvant treatment. This could either be radiotherapy or a re-resection, both having their drawbacks. Radiotherapy has several side effects [13], while a re-resection requires extra operating time and sometimes general anesthesia during a second procedure. Furthermore, problems in localizing the inadequate margin in an already closed wound bed introduce uncertainty about the definitive margin status [14]. 

The existence of (severe) dysplasia in the resection margin adds a different aspect to the discussion of adequate margins. In many patients, the oral SCC develops in an area of (severe) dysplasia, also known as “field cancerization” [15]. There is evidence that when there is residual severe dysplasia after SCC resection, there is a high chance of local recurrence or new primaries [16,17]. There is little consensus about the appropriate treatment in case of severe dysplasia in the resection margin. This could either be CO_2_-laser evaporation or an additional surgical resection [18]. However, surgical resection of all mucosal dysplasia in the case of extensive field cancerization may be an unnecessary overtreatment, potentially leading to increased morbidity. Nevertheless, it is important to differentiate between SCC and (severe) dysplasia in the resection margins, given the varied consequences of residual dysplasia in the resection margins. These consequences encompass differences in locoregional recurrence and the severity of adjuvant treatment. 

In the past decade, an increasing amount of research into intraoperative margin assessment has been conducted that could improve the final margin status. For example, frozen section analysis (FSA) can be used to identify SCC and distinguish it from (severe) dysplasia. This technique uses tissue samples of the wound bed or specimen, which are rapidly assessed for SCC or dysplastic cells through histopathological examination. This allows for the immediate revision of surgical margins, if necessary. However, only 0.1–1% of the specimen and/or wound bed is sampled; therefore, a frozen section may lead to sampling errors, resulting in a low sensitivity for inadequate margins [12,19,20]. Bulbul et al. concluded in a meta-analysis that margin revision indicated by FSA does not lead to better local control [21].

In our center, the application of an intraoperative ultrasound has been investigated for SCC of the buccal mucosa and oral tongue [22,23]. Although it contributed to an enhanced assessment of deep and submucosal margins, it proved difficult to differentiate the tumor and (severe) dysplasia from normal mucosa. Also, intra-operative ex-vivo MRI, which is able to image deep and submucosal margins, has limitations in imaging the mucosal resection plane [24]. However, a margin visualization technique that ensures adequate mucosal margins is equally crucial as achieving adequate submucosal and deep margins. This is preferably a technique that determines the mucosal margin with a high sensitivity for both SCC and dysplasia. 

There are several systematic reviews evaluating margin visualization techniques that may contribute to a higher number of adequate resection margins [25,26,27,28]. However, these reviews discuss only deep margins [28,29] or a combination of deep and superficial margins [26]. Some also include pre-clinical research, research that includes technologies that require sampling of the resection specimen and/or wound bed, or ex-vivo examination of the resection specimen [26,27,29]. 

This systematic review aims to provide an overview of publications evaluating the diagnostic accuracy of recently investigated mucosal margin visualization techniques that aim for adequate mucosal margins, both in the context of SCC and dysplasia. These techniques should be combined with deep margin visualization techniques. We specifically focus on in vivo technologies that are already applied in clinical practice and are suitable for defining the mucosal margin before incisions are made.

## 2. Materials and Methods

This systematic review was conducted following the guidelines outlined in the Preferred Reporting Items for Systematic Reviews and Meta-Analyses (PRISMA) [30] and has not been registered in PROSPERO. 

### 2.1. Eligibility Criteria

The criteria for inclusion were: (1) the study population consisted of patients with a SCC of the head and neck area with a sub-group of oral SCC; (2) an in vivo intraoperatively technique (i.e., directly before the incision, during the resection or directly after the resection) was studied that was able to visualize the entire extent of the mucosal margin during surgery; (3) it aimed to assess or improve resection margin status; and (4) the sensitivity, specificity, positive predictive value (PPV), negative predictive value (NPV), number of free margins, or number of positive margins (in terms of SCC or dysplasia) were mentioned or could be extracted from the publication. 

The criteria for exclusion were: (1) non-clinical studies; (2) publications before 2010; (3) publications that described techniques that only used white light (WL) for tumor/margin visualization, e.g., trans-oral robotic surgery without visual enhancement; (4) publications that described head and neck cancers with <50% oral cancers or without a subgroup analysis of oral cancer in the intervention group; (5) publications that described margin visualization techniques that only work with samples of the resection specimen; (6) publications that described techniques that only identified the presence of SCC or severe (dysplasia) rather than defining a positive or free margin; (7) reviews, case reports, book chapters, editorials, oral presentations, technical notes, and scientific posters; and (8) publications in a language other than English, Dutch, or German.

### 2.2. Search Strategy

A systematic search for relevant publications was performed on PubMed and Embase on 31 August 2023 ( K.J.d.K.). The main focus was to find margin visualization techniques that helped the surgeon identify adequate SCC-free and/or dysplasia-fee margins during surgery. Therefore, search terms focused on the title, abstract, and MeSH terms and included “carcinoma,” all subsites of the oral cavity, and “margins of excision”. The same search terms were used in Embase, but instead of the Mesh terms, the “explode function” was used. Records predating 2010 were excluded from the search based on the assumption that techniques emerging before 2010 lack clinical relevance in the absence of subsequent publications after 2010. The search syntax is shown in Supplementary Materials.

De-duplication was conducted using the method described by Bramer et al. in EndNote (Version 19.3.3, Clarivate Analytics, Philadelphia, PA, USA) [31]. Afterwards, data were exported to Rayyan QCRI (Hamad Bin Khalifa University, Al Rayyan University, Qatar). Two of the three screening authors (C.M.A, K.J.d.K, R.N.) independently assessed the relevance of all titles and abstracts based on the predetermined inclusion and exclusion criteria. Consensus was reached through discussion. Two screening authors (C.M.A, K.J.d.K) reviewed the full texts to determine inclusion or exclusion. Additionally, a reference and citation check was conducted on the selected publications to ensure comprehensive coverage of the entire field of interest. 

### 2.3. Data Extraction

The information extracted from the included publications included the following: year of publication, study methodology (i.e., intervention vs. control or diagnostic accuracy test), sort of index tests (i.e., margin visualization technique), sort of reference-standard (i.e., frozen section analysis or final histopathology), consistency of the cohort (i.e., types of SCC), number of included tumors and/or margins, safety margin distance around the SCC and/or (severe) dysplasia visible under white light safety margin around the area showing positive for the index test, immediate revision based on imaging modality, use of FSA (and whether it was guided by the technique), definition of histopathological positive margin and number of histopathological free margins. 

**Figure 1:**
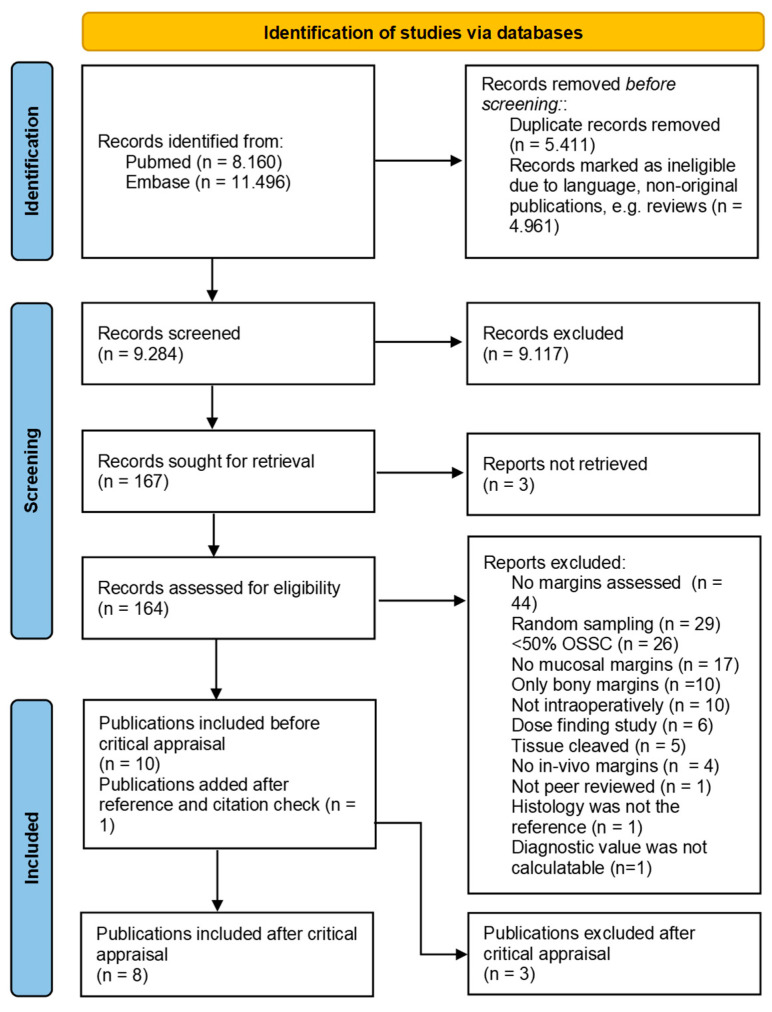
Prisma chart for inclusion and exclusion of publications. From: Page, 2021 [30].

Areas that were indicated by the index test as positive and showed a SCC and/or (severe) dysplasia in that area during the histopathological examination were considered “true positive”(TP), and in the case that no SCC and/or dysplasia was found, “false positive”(FP). Areas beyond the positive index test were considered negative (Figure 1). Depending on whether or not this index-negative area showed SCC and/or (severe) dysplasia, it was deemed false negative (FN) or true negative (TN), respectively. We registered when these variables were determined per resected specimen (specimen-based) or with multiple FSA samples per specimen (sample-based).

If possible, sensitivity (TP cases divided by positive cases according to histopathology), specificity (TN cases divided by negative cases according to histopathology), positive predictive value (PPV) (TP divided by positive cases according to the index test), and negative predictive value (NPV) (TN divided by negative cases according to the index test) were calculated. This was performed, if possible, for the detection of (1) SCC only, (2) SCC in combination with severe dysplasia, and (3) SCC in combination with all types of dysplasia. 

### 2.4. Critical Appraisal

Two screening authors (CA, KK) separately critically appraised the included publications using the Quality Assessment of Diagnostics Accuracy Studies (QUADAS-2) tool [32]. Elements making part of the following categories were assessed to score the risk of bias: (1) ‘patient selection’: a consecutive cohort of patients had been used, the optional control cohort was relevant, and inappropriate exclusions had been avoided; (2) ‘index-test’: the index test was interpreted without knowledge of the reference-standard; (3) ‘reference-standard’: the reference-standard was the final histopathology and the pathologist was blinded for the index test; (4) ‘flow and timing’: the reference-standard and index test were executed equally in each patient and all included patients were analyzed. Applicability was evaluated on the following categories by their elements: (1) ‘patient selection’: oral SCC of both small (T1-T2) and large (T3-T4) tumors were included; (2) ‘index-test’: there was a definition of a positive index, i.e., it used an observer-independent cutoff value and the needed devices and or doses had been described; (3) ‘reference-standard’: a clear definition of a positive margin was given and the reference-standard (i.e. final histopathology) was not affected by additional frozen sections that were not indicated by the margin visualization technique. All items were scored as sufficient: 2 points, unclear: 1 point, or bad: 0 points. The score for each category was determined by summing the points and then dividing the total by the number of items. Overall scores were categorized as ‘insufficient’ within the range of 0–0.5, ‘intermediate’ within the range of 0.6–1.4, and ‘sufficient’ within the range of 1.5–2.0. 

## 3. Results

### 3.1. Search Strategy and Article Selection

The search revealed 19,656 citations (Figure 1). After removing duplicates and records that were marked ineligible because of language (e.g., non-English, non-Dutch, or non-German) or not being an original journal paper (e.g., conference abstract, review, book chapter), 9284 records remained and were screened on title and abstract, leading to 164 records that were screened full text. Eventually, ten records were included and used for the reference standards and citation checks. This led to one additional inclusion, resulting in eleven articles considered eligible for this review. 

### 3.2. Critical Appraisal

An overview of the critical appraisal can be found in Table 1. Considering the risk of bias, none of the studies had a risk of bias for the category ‘index-test’. For ‘patient selection’ and ‘flow and timing’, an intermediate risk of bias was found. Regarding the category ‘reference-standard,’ Baj et al. [33] and Sun et al. [34] scored insufficiently since only FSA or small samples were used to determine diagnostic accuracy, and no final histopathology was used.

Considering applicability, only two studies scored sufficiently in the category ‘patient selection’; Baj et al. [33] and Sun et al. [34] included both early and advanced-stage oral SCC. Durham scored insufficiently for this category, as they did only include small (T1 and T2) tumors or “high grade lesions” defined as dysplasia or in situ carcinoma. 

Tirelli et al.’s 2019 study [43] scored insufficiently for the category ‘index-test.’ They did not clearly define a positive index test while using narrow-band imaging (NBI), possibly because the validation of the NBI technique was not the primary goal of this study. Other studies thoroughly described the definition of a positive index test. However, their description was still observer-dependent and subjective, leading to an ‘intermediate’ score.

Both studies of Morikawa et al. [35,37] scored insufficiently for the category ‘reference-standard’, considering applicability. Both studies did not give a clear definition of a “positive” margin. Moreover, they applied frozen sections in addition to their margin visualization technique but did not discriminate the contribution of the FSA-indicated revisions from the margin visualization technique to the frequency of free margins. The latter issue was also the case for the 2019 study of Tirelli et al. [43]. However, they gave a clear definition of a positive margin. Therefore, they scored ‘intermediate’ for this category.

The fact that the studies of Morikawa et al. [35,37] and Tirelli et al. from 2019 [43] did not discriminate the contribution of the FSA-indicated revisions from the margin visualization technique made it impossible to determine the diagnostic accuracy of the margin visualization technique. Therefore, these studies were excluded from further analysis (Figure 1). Despite other studies scoring ‘insufficient’ on other categories as well [33,34,36], we decided to evaluate their margin visualization technique in this systematic review since it was possible to determine their diagnostic accuracy. This left eight studies for final evaluation. An overview of all studies and their methods of conducting their research can be found in Table 2.

### 3.3. Margin Visualization Techniques

Two included studies investigated autofluorescence [34,36]. Two studies assessed iodine staining [38,39]. Four included studies analyzed NBI [33,40,41,42]. 

**Figure 2:**
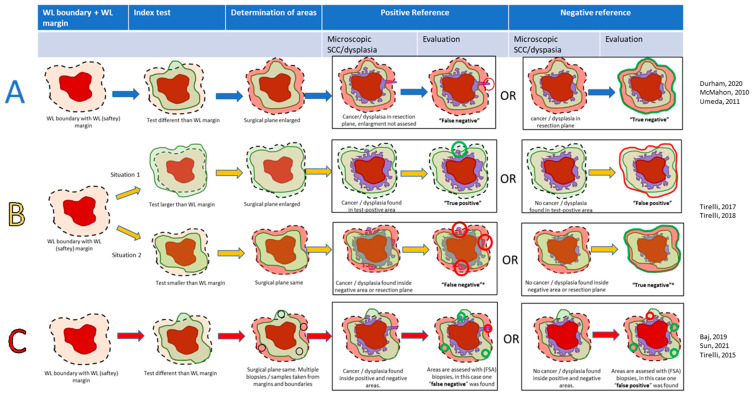
Scheme of the analyzed methods in this review: interventional study (method (**A**), blue arrows), interventional with diagnostic accuracy (method (**B**), yellow arrows), and diagnostic accuracy (method (**C**), red arrows). Dotted line: planned resection margin, which may be changed by the index-test in case of methods (**A**,**B**), light green: positive index-test. Light red: negative index-test (area outside positive area). Dark red: macroscopic tumor. Purple: microscopic tumor or (severe) dysplasia. Bright red: false tests. Bright green: true tests. * In type B studies, if the positive-index test was as large as the WL-safety margin, the specimen was denoted as ‘negative’ [33,34,36,38,39,40,41,42].

In general, the methodology of all studies could be categorized as follows (Figure 2):

- Method A: Interventional studies (with or without a WL-safety margin control group). Surgical margins were enlarged when the index-positive area exceeded the WL-safety margin. SCC and/or dysplasia determined the TN or FN in the index-negative areas surrounding the index-positive areas. Index-positive areas were not analyzed; hence, only the NPV could be calculated. Three studies used this methodology [36,38,39].
- Method B: Interventional studies with diagnostic accuracy. In these studies, the index test was either smaller or larger than the WL-safety margin, and a specimen was either considered index test negative (index ≤ WL) or positive (index > WL). Tumors were excised according to the largest area. Histopathology determined the diagnostic accuracy in these areas. In contrast to Method A, the TP and FP could also be evaluated. In case the index-positive area was as large as the WL-safety margin, the case was considered negative. Two studies used this methodology [41,42].
- Method C: Diagnostic accuracy studies. In these studies, all tumors were excised according to the WL-safety margin. Index-positive areas extending beyond the WL-safety margin were sampled and assessed on the TP or FP. Areas not extending further than the WL-safety margin were also sampled, indicating either the TN or FN. The overlap between the WL-safety margin and positive index test was considered a plausible situation, in contrast to ‘Method C’. Three studies used this methodology [33,34,40].

#### 3.3.1. Autofluorescence

Autofluorescence is one of the multiple imaging techniques that use the fluorescent properties of certain biomaterials. These materials can be excited by absorbing light of a particular wavelength and subsequently emitting this light by a different wavelength. These wavelengths are visible using fluorescence cameras. Instead of external contrast agents with fluorescent properties, autofluorescence margin visualization techniques use the fluorescent properties of biomaterials found within the body, especially those of collagen crosslinks and flavin adenine dinucleotide. When blue light (wavelength 400–460 nm) is absorbed by normal tissue, it subsequently re-emits light that appears green when observed through a filter. Abnormal tissue, such as neoplastic, dysplastic, and inflammatory tissue, cannot be excited and does not emit green light but appears brown through the filter [44]. These so-called fluorescence visualization loss (FVL) areas can be delineated with a certain margin to obtain the free margin status. 

One interventional study by Durham et al. (‘Method A’) performed a randomized controlled trial with a minimal 10 mm WL-safety margin and minimal 10 mm FVL-safety margin [36]. They included OSCCs (n = 261) and high-grade lesions (i.e., severe dysplasia, n = 182). This study only reported the “first-pass margin”; margins found “positive for severe dysplasia or greater histopathologic change” and thus seemed not to make a difference between SCC and (severe) dysplasia. Additional revisions were possibly conducted but not described, resulting in an unknown number of free margins in final histopathology. The NPV of their test cohort (70%) was similar to that of their conventional cohort (70%).

One study by Sun et al. performed a ‘Method C’ study on autofluorescence by applying a demarcation on the boundary of the FVL-positive area [34]. They included only SCC patients. Then, they resected the specimen with a 15–20 mm WL-safety margin. In all cases, the FVL-positive area fell within this WL-safety margin. Samples (n = 126) collected from random locations between the FVL-based demarcation and resection plane were assessed on the frequency of SCC and/or (severe) dysplasia beyond the FVL-positive area. For SCC in the samples, this frequency was 0% (NPV 100%). For severe dysplasia, the frequency was 18% (NPV 82%). For mild dysplasia, the frequency was 21%. As no moderate dysplasia was found, for all types of dysplasia, the frequency was 39% (NPV was 61%). 

An overview of autofluorescence’s diagnostic accuracy can be found in Table 3.

#### 3.3.2. Iodine Staining

Iodine staining has been widely used for the detection of intraepithelial neoplasia of the esophagus but can also be used to detect oral SCC and dysplasia [38]. Iodine stains healthy tissue and creates an iodine unstained (IU) area on the SCC or dysplastic tissue. Similar to autofluorescence, an IU-safety margin around the IU boundary can be applied to achieve free margin status. Only two interventional studies using ‘Method A’ were included that assessed this method [38,39].

One study by McMahon et al. used a 10 mm WL-safety margin and a 0 mm IU-safety margin [38]. They compared their prospective iodine-guided surgery cohort, consisting of 40/50 (80%) patients with oral SCC, with a retrospective WL-guided surgery cohort, consisting of 42/50 (84%) patients with oral SCC. They found no SCC-positive margins in the iodine-guided cohort (NPV of 100%) and 2/50 (4%) SCC-positive margins (NPV of 96%) in the WL-guided surgery cohort. They found 1/50 (2%) severe dysplasia and 1/50 (2%) other types of dysplasia in the iodine-guided cohort and 1/50 (2%) severe dysplasia and 13/50 (26%) other types of dysplasia in the WL-guided cohort. The NPV for dysplasia (all types) was 96% in the iodine cohort and 68% in the WL-guided cohort.

One single-arm study by Umeda et al. used a 10 mm WL-safety margin and a 5 mm IU-safety margin in a cohort consisting of 93 SCCs of the tongue [39]. They found in their retrospective cohort that only 1/93 (1%) of the patients had SCC-positive mucosal margins, leading to an NPV of 99% for SCC. They found that 6/93 (6%) of the patients had mucosal margins positive for mild dysplasia, leading to an NPV of 94%. The NPV for dysplasia and SCC combined was 86/93 (92%). 

Both studies suggest that using iodine is excellent for determining mucosal safety margins and results in most margins free of SCC and dysplasia. The NPV for SCC and dysplasia (all types) of McMahon et al.’s iodine-guided surgery cohort [38] suggest that iodine has the potential to rule out moderate and mild dysplasia in the resection margin when compared to the results of the WL-guided surgery cohort. However, these results assessed the impact of iodine staining in conjunction with the IU-safety margin, lacking specific information on the sensitivity and specificity of the IU area alone.

An overview of iodine’s diagnostic accuracy can be found in Table 4.

#### 3.3.3. Narrow Band Imaging

NBI is a technique where the surgical field is illuminated by WL, but the reflection is filtered to only two specific wavelengths (415 and 540 nm) that enhance the visualization of the capillary bed and the intrapapillary loop pattern in the superficial mucosa [41]. Changes in the architecture of the capillaries may indicate SCC or dysplasia in the oral cavity. NBI can be applied to an endoscope and is therefore applicable in surgeries of both the oral and oropharyngeal mucosa. Two ‘Method B’ [41,42] studies and two ‘Method C’ [33,40] assessing NBI were included.

The two ‘Method B’ studies were conducted by Tirelli et al.: one from 2017 [41] and one from 2018 [42]. In their 2017 study, Tirelli et al. [41] evaluated a cohort that consisted of 20/31 (65%) oral SCC patients. In 28/31 (90%) of the patients, the safety margin was expanded, as the NBI-positive area was larger than the 15 mm WL-safety margin, which was considered to be a positive index test. Of these 28 cases, 20 were TPs (i.e., SCC and/or dysplasia of all types found in the extended margin), and 8 were FPs (i.e., no SCC and/or dysplasia of all types found in the extended margin). In 2/31 cases (7%), the NBI-positive area was similar to the 15 mm WL-safety margin, and in only 1/31 (3%) cases, the NBI-positive area was smaller than the 15 mm WL-safety margin. For these three cases, an extension of the safety margin was not needed. Hence, there were three negative index tests, although the authors only reported the presence of SCC and/or dysplasia (all types) in the case with the smaller NBI margin, resulting in one TN case and no FN case. These results yielded a sensitivity of 100% (CI: 83–100%), specificity of 11% (CI: 0–29%), PPV of 71% (CI: 66–76%), and NPV of 100% (CI: 3–100%), for SCC and dysplasia (all types). 

Tirelli et al.’s 2018 study [42], used exactly the same method as their 2017 study [41] in a cohort of 39/61 (64%) oral SCC patients. Of 43/61 (70%) cases, an extension of the safety margin was needed, as the NBI-positive area was larger than the 15 mm WL-safety margin (i.e., positive index test). Of these 43 cases, 34 were TPs (i.e., SCC and/or dysplasia of all types in the extended margin), and 9 were FPs (i.e., no SCC and/or dysplasia of all types in the extended margin). In 18/61 (30%) cases, no extension of the safety margin was indicated by NBI, i.e., a negative index test. Sixteen of these 18 cases were TNs, and 2 were FNs. These results yielded a sensitivity of 94% (CI: 81–99%), specificity of 64% (CI: 42–82%), PPV of 79% (CI: 69–87%), and NPV of 89% (CI: 67–97%) for SCC and dysplasia (all types).

Two ‘Method C’ studies analyzed the diagnostic accuracy of NBI, one by Baj et al. [33] and one by Tirelli et al. from 2015 [40]. Baj et al. [33] assessed a cohort that consisted entirely of oral SCC patients (n = 16). They varied the distance of the WL-safety margin between 15 and 20 mm and took three to eight biopsies per specimen, situated at the border of the NBI-positive areas and of those of the WL-safety margin. After the FSA examination, biopsies were classified as positive or negative for “SCC or dysplasia (all types)”. The authors did not discriminate SCC from dysplasia. Three TPs, 5 FNs, 14 FPs, and 32 TNs were found to yield a sensitivity, specificity, PPV, and NPV of 38% (CI: 9–76%), 70% (CI: 54–82%), 18% (CI: 7–37%), and 86% (CI: 78–92%), respectively. Contours of the NBI-positive areas were within the WL-safety margin in 50% of the cases. 

Tirelli et al. [40] found in their ‘Method C’ study from 2015 that the 15 mm WL-safety margin was surrounded by a NBI-positive area in every case. This contrasts with the results from Baj et al. [33], who reported this situation in only 50% of the cases. They performed an FSA in the NBI-positive area and extended the surgical margin according to the NBI in case dysplasia or a SCC was found. In every case, SCC and/or dysplasia were found beyond the 15 mm WL safety margin. For SCC only, it resulted in 12 TPs, 0 FNs, 4 FPs, and 0 TNs cases, yielding a PPV of 75%, a sensitivity of 100%, and a specificity of 0%, but no calculable NPV. For SCC and dysplasia (all types), it resulted in 16 TPs, 0 FNs, 0 FPs, and 0 TNs cases, yielding a PPV of 100%, a sensitivity of 100%, but no calculable specificity or NPV. Although the safety margins were enlarged when FSA confirmed TP, there was still one specimen with SCC-positive margins (6%) and one specimen with margins positive for dysplasia. 

NBI is the only assessed technique in this review, of which three out of four studies report both a calculable PPV, NPV sensitivity, and specificity. However, a wide variety of methods are employed to obtain these outcome measurements across the studies. 

An overview can be found in Table 5.

## 4. Discussion

This systematic review highlights techniques that try to define the optimal mucosal surgical resection margins in the treatment of oral SCC. The demarcation of the mucosal surgical margin is an essential part of oral cancer surgery because it serves as a critical reference point for the surgeon to achieve tumor-free (i.e., ≥5 mm) histopathological margins in all dimensions. In the past years, more attention has been given to margin visualization techniques that aid the surgeon in estimating the deep extension of the tumor. Although several systematic reviews assess these techniques, to our knowledge, no reviews specifically illuminate the currently evaluated techniques that enhance the demarcation of the mucosal surgical margin in oral cancer surgery. This systematic review tries to fill in this gap in the literature. 

During the setup of this review’s methodology, we attempted to assess the visualization techniques by their diagnostic value in identifying positive margins and free margins as defined by the Royal College of Pathologists [10], i.e., <1 mm and ≥5 mm SCC free margins, respectively. However, no studies were found assessing the diagnostic accuracy for close margins with respect to SCC (1–5 mm). Instead, all studies seemed to focus on the presence of SCC or (severe) dysplasia in the resection plane, some of them not making a difference between the SCC or (severe) dysplasia. Indeed, several studies suggest that residual dysplasia has similar effects on disease-free survival as close margins [16,17]. Hence, dysplasia is preferably resected during SCC surgery. However, when compared to residual dysplasia, residual SCC has a far greater impact on patient survival. Moreover, residual SCC requires adjuvant treatments (radiotherapy or re-resections) with higher risks and complication rates compared to CO_2_-laser evaporation for residual dysplasia [12,13,18,45]. Unfortunately, none of the included studies discussed the incidence of close mucosal resection margins (1–5 mm free of SCC), and some did not differentiate between SCC and (severe) dysplasia in the resection plane. 

This systematic review included studies to examine the benefits of margin visualization techniques in a surgical context. Consequently, studies that specifically reported negative or clear margins were included, while those that solely assessed the presence of tumors were not included. As a result, three of the selected studies primarily consisted of interventional research (‘Type A’ studies) [36,38,39]. These studies do not generate a positive index test, as the surgical goal is to achieve a negative index test. Therefore, calculating a meaningful sensitivity, specificity, or PPV is impossible. For these studies, we cannot determine whether the implementation of these margin visualization techniques will result in potential over-treatment, i.e., unnecessary wide resection margins. Nevertheless, although strongly dependent on the incidence of histologically positive margins, the NPV indicates the effectiveness of the margin visualization technique for the resection of SCC and/or dysplasia. 

In one ‘Method C’ study that investigated autofluorescence, conducted by Sun et al., NPV was the only measurement for diagnostic accuracy that could be reported, as the authors found that all FVL-areas were smaller than the 15–20 mm WL-safety margin (i.e. negative-index-test) [34]. This means that also, for this study, no valuable comparison between the diagnostic accuracy for identification of SCC-positive margins and dysplastic-positive margins was possible. While the authors used the WL-safety margin during the resection, their NPV of 100% for SCC in the resection plane showed that if an FVL-safety margin had been used, no SCC would have been found in the resection plane. However, for severe dysplasia and all types of dysplasia, the NPV would have been 28% and 39%, respectively. The presented numbers are comparable with the multicenter randomized controlled trial of Durham et al. [36], who found severe dysplasia in the resection plane in 30% when autofluorescence guidance was used. The frequency of positive margins and 5-year local recurrence were not lower in the autofluorescence-guided cohort when compared to the WL-guided cohort. According to the authors, these unexpected results were most likely caused by the relative inexperience in using autofluorescence of the participating centers outside the coordinating center. In the studies by Morikawa et al., larger FVL-safety margins were used (in combination with iodine), yet there was a considerable amount of FSA-positive rate for SCC and/or dysplasia (all types), namely 19% and 18%. 

Two interventional (‘Method A’) studies using iodine-guided surgery reported a positive margin rate per specimen. McMahon et al. [38] compared an iodine-guided cohort with a WL-guided control cohort. They only found a significant difference between both cohorts when all types of dysplasia were considered positive (96% in the iodine-guided cohort vs. 68% in the WL-guided control cohort), which suggests that iodine-guided surgery makes the most difference in the detection of moderate or mild dysplasia. Umeda et al. found comparable results and reported no local recurrence in their single-arm study [39]. 

All studies examined NBI guidance assessed dysplasia (all types) in the resection plane, but only several studies did this specifically for SCC and/or severe dysplasia [40,41,42]. Baj et al. [33] reported a lower sensitivity for SCC and dysplasia (combined) in the resection plane (38%) compared to Tirelli et al.’s studies, which ranged from 94% to 100%. The reduced TP rate in Baj et al. may be subject to their sampling strategy—taking samples from the borders of NBI-positive areas, unlike Tirelli et al., who sampled within NBI-positive areas. In the diagnostic accuracy study (‘Type B’) of Tirelli from 2017, only one negative index test was found [41]. Interestingly, their subsequent study showed a much higher number of negative index tests [42]. This figure might have been the result of a learning curve.

Based on the included studies, it is impossible to determine whether autofluorescence, iodine guidance, or NBI is more accurate than WL-guided surgery to determine a safe surgical mucosal margin and also in terms of distinguishing (severe) dysplasia from SCC. There are several reasons. 

Firstly, there is a high variety in the definition of a positive reference-standard dysplasia: i.e., SCC, SCC in combination with severe dysplasia, or SCC in combination with all types of dysplasia in the resection plane. Several studies do not differentiate between SCC and (severe) dysplasia.

Secondly, the index tests of all studies were not designed to distinguish (severe) dysplasia from SCC but rather tissue that was divergent from normal mucosa. For autofluorescence, neoplastic, dysplastic, and inflammatory tissue all show FVL [44]. Staining with Lugol’s iodine is based on the fact that iodine is glycophilic and does not bind to cells that lack glycogen, leading to iodine unstained areas. However, SCC and dysplasia both lack glycogen; therefore, Lugol’s iodine cannot differentiate between tissue types [38]. Finally, NBI is based on detecting alternations in the interpapillary capillary loops, which can underlie histopathologic changes, but this accounts for both SCC and all types of dysplasia [40]. 

Thirdly, all studies are possibly subject to high inter and intra-observer variability, requiring expertise and experience to achieve a sufficient diagnostic value. None of the studies presented a clear cutoff value to define a positive or negative index test. In the studies of Tirelli et al., NBI experts needed to be consulted to determine the NBI-safety margin, suggesting that finding alterations in the intrapapillary capillary loop patterns is difficult. Hence, they have found a variety of diagnostic accuracies [40,41,42]. 

Fourthly, the included studies have a relatively small number of included patients or conducted retrospective studies. Only Durham et al. [36] conducted a randomized clinical trial and may pose the highest level of evidence that autofluorescence guidance does not influence obtaining more adequate margins or more local control than WL guidance. However, the inexperience of certain observers and the surgeons’ awareness of obtaining adequate margins in the WL-guided control cohort might have influenced the results. 

Lastly, in most studies, only the NPV could be calculated. The sensitivity, specificity and PPV remain unknown for autofluorescence and iodine guidance. The lack of this information complicates the assessment of their potential impact on a “tailored-made” approach. Without these data, it remains unclear how the adjustment of the safety margin around a positive index test could affect surgical margins, either by expanding or reducing them. Only two studies suggested that NBI guidance could lead to more tailored-made resections. Tirelli et al. have shown a specificity of 64%, meaning that 64% of the margins positive for SCC or dysplasia were rightfully made smaller if only a resection plane free of SCC or dysplasia is considered acceptable [42]. For Baj et al., this number was 70% [33].

There are several other margin visualization techniques that could lead to new insights when investigated in a surgical setting. Optical coherence tomography (OCT), for instance, works essentially in the same manner as an ultrasound but uses light instead of sound waves. Because of the short wavelength of light, its penetration depth is not more than 0.5 mm for mucosa, but it can provide highly detailed images [46]. At the moment, the setup of OCT devices mostly does not allow intraoral assessment [47]. In one study by Sunny et al. [48], a hand-held OCT device was introduced for intraoral use. The authors captured images of multiple zones around the tumor and compared them with the histopathological report. The observers of the OCT data were blinded for the surgical procedure. They found that OCT was able to detect SCC inside the tumor and the area around the visible tumor with a sensitivity and specificity of 100%. For dysplasia, the sensitivity and specificity were 93% and 69%, respectively. The study was not included in this review because of the limited field of view of the device [48]. Further development is needed to eventually assess the whole mucosal part of a tumor with OCT.

Other fluorescence-guided techniques exist besides autofluorescence. Contrast-agent-based fluorescence uses a near-infrared fluorescent label for SCC-specific antigens, such as cetuximab [49] or panitumumab [50]. This technique can be used intra-orally but mostly to check the wound bed on any residual fluorescent signal [49]. The scope of most studies researching this technique is an ex vivo assessment of the resection specimen. FSA biopsies can be taken from the spot with the highest fluorescent signal and analyzed to determine whether this margin is close or positive. If not, it may suggest that the other fluorescent spots on the specimen are free margins as well [50]. One major advantage of this technique is that it can produce objective values for the index test, i.e., the signal-to-background ratio of the fluorescence signal, which eliminates inter-observer dependence, as presented by de Wit et al [49]. As autofluorescence does not yield significant improvements in obtaining mucosal margins when compared to WL-guided surgery, it would be interesting to investigate the impact of contrast-agent-based fluorescence on mucosal margins in randomized control trials, following a similar setup as Durham et al. [36].

Apart from iodine staining, staining with toluidine blue has also been researched. However, the studies of concern [51,52] stained the resection specimen, but only after the resection was completed. These studies concluded that this stain is highly sensitive to SCC in the resection margins but has a low PPV. Kerawala et al. [53] performed a study on the intra-oral use of toluidine blue as a margin visualization technique, but this study was also not included since it was published before 2010. They concluded that Toluidine blue is a suitable adjunct in identifying invasive tumors but has no benefit in identifying dysplastic tissue at the surgical margins. Unfortunately, their findings did not result in further research on the intraoperative application of Toluidine blue in the past decade.

Several limitations should be acknowledged in this review. Firstly, the inclusion of various methodologies (such as ’Method A’) and diverse outcome measures (including diagnostic accuracy for both ’SCC and dysplasia’ or ’SCC alone’) poses a challenge in assessing potential publication bias. This complexity makes it difficult to employ standard methods like funnel plots or Egger’s test for a comprehensive evaluation. Secondly, as some included articles have the same author (i.e., Tirelli et al.) and were published within four years while assessing the same technique, it cannot be ruled out that there may be some overlap between the described cohorts. However, evidence is lacking to confirm or refute this possibility. 

We suggest that future studies on margin visualization techniques should focus more on the differentiation between (severe) dysplasia and SCC. Moreover, the evaluation of diagnostic accuracy should go beyond the goal to achieve only a negative index test. Ideally, a setup presented by Sunny et al. [48] would give a broader insight into the diagnostic accuracy for SCC and severe dysplasia. Independent observers designated the images obtained from the OCT device as “normal,” “potentially malignant,” and “malignant”. This was conducted at different zones from the tumor border, which makes it feasible to determine the diagnostic accuracy for SCC and/or dysplasia in the resection plane but also for close margins (SCC at 1–5 mm from the resection plane). If technically possible, the margin visualization technique should also be as inter-observer-independent as possible. An example is the signal-to-background ratio-based fluorescence of de Wit et al. [49], where the author used an objective value to determine tumor presence. 

## 5. Conclusions

Three margin visualization techniques for oral SCC have been reviewed in a pre-incision surgical setting to determine a safe mucosal margin demarcation: autofluorescence, iodine staining, and NBI. Most of these studies did not assess the frequency of free margins (≥5 mm) but only the presence of dysplasia and SCC in the resection plane. Apart from fluorescence, the margin visualization techniques found a wide variety in diagnostic accuracy, possibly due to learning curves and inter- or intra-observer variability. Autofluorescence guidance seems to make no difference in obtaining better margins than WL guidance. However, contrast-agent-based autofluorescence might be more effective, and testing this technique in large randomized controlled trials is advisable. We also recommend continuing to investigate iodine and NBI-guided surgery in more extensive cohorts, with a larger focus on differentiation between (severe) dysplasia and SCC, as the consequences of the treatment of residual dysplasia and SCC are highly different. Apart from reporting the treatment effect of the technique in terms of margins ‘free from SCC and (severe) dysplasia’, the presence of close (1–5 mm) or free (≥5 mm) margins should be reported as well, according to the standard guidelines. Finally, we recommend a larger focus on actual diagnostic accuracy rather than treatment effect only. This strategy would allow for determining a meaningful sensitivity, specificity, and PPV, in addition to negative predictive value (NPV). Such an approach will lead to a better understanding of the value of these techniques.

## Figures and Tables

**Table 1:** Critical appraisal of included studies after text screening.

	Risk of Bias				Applicability		
	Patient Selection	Index Test	Reference	Flow and Timing	Patient Selection	Index Test	Reference
Morikawa, 2019 [35]	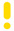	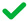	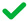	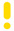	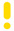	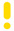	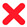
Durham, 2020 [36]	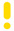	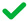	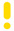	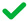	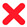	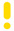	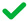
Sun, 2021 [34]	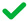	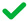	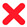	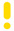	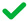	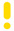	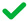
Morikawa, 2023 [37]	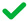	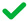	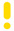	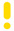	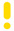	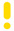	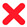
McMahon, 2010 [38]	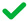	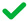	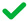	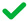	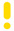	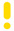	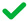
Umeda, 2011 [39]	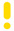	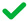	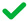	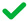	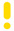	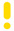	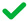
Tirelli, 2015 [40]	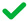	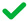	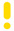	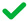	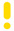	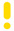	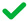
Tirelli, 2017 [41]	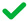	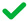	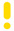	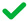	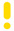	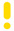	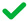
Tirelli, 2018 [42]	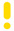	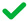	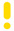	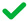	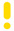	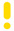	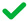
Tirelli, 2019 [43]	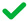	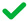	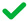	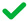	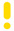	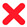	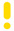
Baj, 2019 [33]	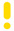	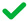	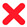	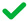	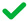	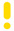	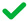

Green check marks: ‘sufficient.’ Yellow exclamation marks: ‘intermediate.’ Red cross marks: ‘insufficient.’

**Table 2:** Methods from included studies.

Author	Method	Technique	Cohorts/Survival Analysis	No. of Tumors/No. of Margin Samples	Consistency	Demarcation of Safety-Margin	Positive Margin Defined by Publication	Definition of Free Margin by Authors	Acquisition Time	Technique Influence on the Final Histopathology	Free Margin Status on the Final Histopathology
Durham, 2020 [36]	A	Autofluorescence	Autofluorescence (R) vs. WL-guided surgery (R)/survival analysis	443	Autofluorescence: 277 OSCC + HGL, control: 216 OSCC + HGL	At least 10 mm from the boundary of the WL-positive and FVL-positive areas	“Positive margin for severe dysplasia or greater histologic change”.	Not given	Undefined	Yes	Undefined, only the “first pass margin” is given, defined as the positive margin before re-resections are taken from the tumor bed.
Sun, 2021 [34]	C	Autofluorescence	Diagnostic accuracy of autofluorescence (P)	30/126	30 OSCC	15-20 mm from the boundary of the WL-positive area, no resections based on the FVL-positive area	Carcinoma in situ, invasive carcinoma, and severe dysplasia in mucosal samples	Normal epithelium in mucosal samples	Undefined	No	Undefined, only margins within random samples were reported
McMahon, 2010 [38]	A	Iodine	Iodine (P) vs. WL-guided surgery ^®^	100	Iodine: 40 OSCC and 10 OPSCC, control: 42 OSCC, 8 OPSCC	10 mm from the boundary of the WL-positive area and 0 mm from the IU-positive areas were included	“Intraepithelial neoplasia in mucosal margin”.	“Mucosal margins free from dysplasia, carcinoma in situ and invasive carcinoma”	Undefined	Yes	Iodine: 96% (48/50), WL 96% (34/50)
Umeda, 2011 [39]	A	Iodine	Iodine (R)/Survival analysis.	93	93 OSCC of the tongue	At least 10 mm from the boundary of the WL-positive areas and at least 5 mm from the boundary of the IU-positive areas	“Positive for SCC” or ‘’positive for dysplasia”.	SCC ≥ 1 mm from the deep or mucosal margin	Undefined	Yes	81/93 (87%)
Tirelli, 2015 [40]	C	NBI	NBI (P)	16	8 OSCC, 8 OPSCC	15 mm from the boundary of the WL-positive areas and including the boundary of the NBI-positive areas.	SCC < 0.1 mm from the mucosal margin	SCC > 3 mm from the mucosal margin	5 min	Yes, technique-directed FSA did not influence diagnostic accuracy	94% (15/16)/yes
Tirelli, 2017 [41]	B	NBI	NBI (P)	31	20 OSCC, 11 OPSCC (of 2 the result of the reference test was not clear)	15 mm from the boundary of the WL-positive areas and including the boundary of the NBI-positive areas.	SCC < 0.1 mm from the mucosal margin	SCC > 3 mm from the mucosal margin	5 min	Yes, FSA, in addition to the technique, did not influence diagnostic accuracy	77% (24/31)/yes
Tirelli, 2018 [42]	B	NBI	NBI (P)	61	39 OSCC, 22 OPSCC	15 mm from the boundary of the WL-positive areas and including the boundary of the NBI-positive areas.	SCC < 0.1 mm from the mucosal margin	SCC > 3 mm from the mucosal margin	5 min	Yes, FSA, in addition to the technique, did not influence diagnostic accuracy	85% (52/61)/yes
Baj, 2019 [33]	C	NBI	NBI (P)	16/ 88	16 OSCC	15-20 mm from the boundary of the WL-positive area, no resections based on the NBI-positive area	Tumor or dysplasia in the FSA biopsy	No tumor or dysplasia in FSA biopsy	Undefined	No	Undefined, only FSA biopsy status reported

WL: white light, NBI: narrow band imaging, FVL: fluorescence visualization loss, OSCC: oral squamous cell carcinoma, OPSCC: oropharyngeal squamous cell carcinoma, SCC: squamous cell carcinoma, R: retrospective, P: prospective, FSA: frozen section analysis, HGL: high-grade lesions.

**Table 3:** Results from studies about autofluorescence.

Author	Evaluation	Reference Results Based on	Test Positive/Negative	Ref Positive/Negative	NPV SCC (Test/WL Control Group)	NPV SCC + Severe Dysplasia (Test/WL Control Group)	NPV SCC + Dysplasia (Test/WL Control Group)	Bias or Concern
Durham, 2020 [36]	Interventional (with WL-guided control group)	Full specimen (OSCC or HGL)	NA/10 mm from the WL-positive area and 10 mm from the FVL-positive area (whichever was wider)	“Severe dysplasia or greater histologic change” in the resection plane on the final histopathology/normal tissue in the resection plane on the final histopathology	Not given	Test: 70% (151/216) Control: 70% (159/227)	Not given	Unknown reason for certain exclusions; patients with small tumors and “High-grade lesions” were included as well.
Sun, 2021 [34]	Diagnostic accuracy	Samples from margin	Sample within the FVL-positive area exceeding the WL-positive area/sample within the FVL-positive area inside the WL-positive area	SCC or dysplasia (all types) in the sample of the FVL-positive area/normal tissue in the sample of the FVL-positive area	100% (126/126)	82% (103/126)	61% (77/126)	126 samples were taken and analyzed from random locations between the boundary of the FVL-positive area and the surgical margin of 30 tumors

WL: white light surgery, FVL: fluorescence visualization loss, FSA: frozen section analysis, NPV: negative predictive value, SCC: squamous cell carcinoma, NA: not applicable.

**Table 4:** Results from studies about iodine.

Author	Evaluation	Reference Results Based on	Test Positive/Negative	Ref Positive/Negative	NPV SCC (Test Group/WL Control Group)	NPV SCC + Severe Dysplasia or (Test/WL Control Group)	NPV SCC + Dysplasia (Test/WL Control Group)	Bias of Concern
McMahon, 2020 [38]	Interventional (with WL-guided control group)	Full specimen	NA/10 mm from the boundary of the WL-positive area, 0 mm from the IU-positive area	Dysplasia (all types)or SCC in the resection plane	Test: 100% (50/50) Control: 96% (48/50)	Test: 98% (49/50) Control: 96% (47/50)	Test: 96% (48/50) Control: 68% (34/50)	None.
Umeda, 2011 [39]	Interventional (no WL-guided surgery control group)	Full specimen	NA/ 10 mm from the boundary of the WL-positive area and 5 mm from the IU-positive area	Dysplasia or SCC in the resection plane	99% (92/93)	Not given, only mild dysplasia in the resection plane	92% (86/93)	None.

WL: white light, FSA: frozen section analysis, NPV: negative predictive value, CIS: carcinoma in situ, IU: iodine unstained, SCC: squamous cell carcinoma, NA: not applicable.

**Table 5:** Results from studies about NBI.

Author	Evaluation	Reference Results Based on	Test Positive/Negative	Ref Positiv/Negative	Sens/Spec Cancer	PPV/NPV Cancer	Sens/Spec SCC + Sdys	PPV/NPV SCC + Sdys	Sens/Spec SCC + Dys (all Types)	PPV/NPV SCC + Dys (All Types)	Bias of Concern
Tirelli, 2015 [40]	Diagnostic accuracy	FSA-samples	The NBI-positive area beyond the 15 mm WL-safety margin/NBI-positive area between the boundary of the WL-positive area and 15 mm WL-safety margin	SCC and/or dysplasia/no SCC and/or dysplasia in the NBI-positive or negative area	Sens: 100% (12/12) Spec: 0% (0/4)	PPV: 75% (12/16) NPV: undefined (0/0)	NA	NA	Sens: 100% (16/16) Spec: undefined (0/0)	PPV: 100% (16/16) NPV: undefined (0/0)	Only the NBI-positive areas were assessed with biopsies, while the NBI-negative areas, (mucosa within the 15 mm W-safety margin, but outside the boundary of the NBI-positive area) did not receive a biopsy. Also, the NBI-positive area seemed too small, since dysplasia and SCC were found in the resection plane. Possible overlap with Tirelli 2017 and Tirelli 2018.
Tirelli, 2017 [41]	Interventional with diagnostic accuracy	Final histopathology	NBI-positive area beyond the 15 mm WL-safety margin/ NBI-positive area between the boundary of the WL-positive area and 15 mm WL-safety margin	SCC and/or dysplasia/no SCC and/or dysplasia in the NBI-positive or negative area	Sens: 100% (12/12) Spec: 6% (1/17)	PPV: 43% (12/28) NPV: 100% (1/1)	Sens: 100% (16/16) Spec: 8% (1/13)	PPV: 57% (16/28) NPV: 100% (1/1)	Sens: 100% (20/20) Spec: 11% (1/9)	PPV: 71% (20/28) NPV: 100% (1/1)	Only one specimen with NBI-negative findings (specimen with the boundary of the NBI-positive area within the WL margin). Of two specimens, the NBI-positive areas were as large as the WL-safety margin, but it was unclear whether the resection planes were free from SCC/dysplasia. Hence, only 29 cases could be evaluated. Possible overlap with Tirelli 2015 and Tirelli 2018.
Tirelli, 2018 [42]	Interventional with diagnostic accuracy	Final histopathology	NBI-positive area beyond the 15 mm WL-safety margin/ NBI-positive area between the boundary of the WL-positive area and 15 mm WL-safety margin	SCC and/or dysplasia/no SCC and/or dysplasia in the NBI-positive or negative area	Sens: 96% (23/24) Spec: 46% (17/37)	PPV: 53% (23/43) NPV: 94% (17/18)	Sens: 93% (28/30) Spec: 52% (16/31)	PPV: 65% (28/43) NPV: 89% (16/18)	Sens: 94% (34/36) Spec: 64% (16/25)	PPV: 79% (34/43) NPV: 89% (16/18)	Possible overlap with Tirelli 2015 and Tirelli 2017.
Baj, 2019 [33]	Diagnostic accuracy	FSA-samples	NBI boundary outside 15–20 mm from the WL boundary/NBI-positive area between the boundary of WL-positive area and 15 mm WL-safety margin	Dysplasia or SCC in FSA biopsy from a positive test situation/no dysplasia or SCC in FSA biopsy	Not given	Not given	Not given	Not given	Sens: 38% (3/8) Spec: 70% (32/46)	PPV: 18% (3/17) NPV: 86% (32/37)	Only small biopsies were taken for certain areas. The WL-safety margin was not from a consistent distance from the WL-positive boundary (varying between 15 and 20 mm).

WL: white light, NBI: narrow band imaging, FSA: frozen section analysis, SCC: squamous cell carcinoma, Sens: sensitivity, Spec: specificity, NPV: negative predictive value, PPV: positive predictive value, Dys: dysplasia, Sdys: Severe dysplasia, NA: not applicable.

## Supplementary Materials

The following supporting information can be downloaded at: https://www.mdpi.com/article/10.3390/cancers16061148/s1”. PubMed search term and Embase search term.
