# Supplementary material for: Intraoperative Techniques That Define the Mucosal Margins of Oral Cancer In-Vivo: A Systematic Review
Source: Cancers (Basel). 2024 Mar 14;16(6):1148. doi: 10.3390/cancers16061148 (PMC10968727; doi:10.3390/cancers16061148)
Supplement: Supplementary file 1 [file cancers-16-01148-s001.zip › cancers-2850140-supplementary.pdf]

Supplementary: PubMed search term: (((("oral cavity"[Title/Abstract] OR "gingiva*"[Title/Abstract] OR "tongue"[Title/Abstract] OR "floor of mouth"[Title/Abstract] OR "head and neck"[Title/Abstract] OR "head neck"[Title/Abstract] OR "buccal"[Title/Abstract] OR "oral mucosa*"[Title/Abstract] OR "oral*"[Title/Abstract] OR "gum"[Title/Abstract] OR "retromolar"[Title/Abstract] OR "alveolar"[Title/Abstract] OR "cheek"[Title/Abstract] OR "maxilla*"[Title/Abstract] OR "mandib*"[Title/Abstract] OR "jaw"[Title/Abstract]) AND ("cancer"[Title/Abstract] OR "cancers"[Title/Abstract] OR "carcinoma"[Title/Abstract] OR "carcinomas"[Title/Abstract] OR "tumor"[Title/Abstract] OR "tumour"[Title/Abstract] OR "tumors"[Title/Abstract] OR "tumours"[Title/Abstract] OR "malignan*"[Title/Abstract] OR "squamous cell carcinom*"[Title/Abstract])) OR ("oral cancer*"[Title/Abstract] OR "OSCC"[Title/Abstract] OR "oral squamous cell carcinom*"[Title/Abstract] OR "HNSCC"[Title/Abstract] OR "oral carcinoma"[Title/Abstract] OR "oral cancers"[Title/Abstract] OR "oral carcinomas"[Title/Abstract] OR "oral malignan*"[Title/Abstract] OR "oral tumor"[Title/Abstract] OR "oral tumors"[Title/Abstract] OR "oral tumours"[Title/Abstract] OR "oral tumour"[Title/Abstract] OR "oral tumor*"[Title/Abstract] OR "oral tumors"[Title/Abstract]) OR "mouth neoplasms"[MeSH Terms]) AND ("border*"[Title/Abstract] OR "margin*"[Title/Abstract] OR "tumor free resection*"[Title/Abstract] OR "margins of excision"[MeSH Terms] OR "thickness"[Title/Abstract] OR "depth of invasion"[Title/Abstract] OR "DOI"[Title/Abstract])) AND (2010:2023[pdat]). **Embase search term:** (('oral cavity':ti,ab,kw OR 'gingiva*':ti,ab,kw OR 'tongue':ti,ab,kw OR 'floor of mouth':ti,ab,kw OR 'head and neck':ti,ab,kw OR 'head neck':ti,ab,kw OR 'buccal':ti,ab,kw OR 'oral mucosa*':ti,ab,kw OR 'gum':ti,ab,kw OR 'retromolar':ti,ab,kw OR 'alveolar':ti,ab,kw OR 'cheek':ti,ab,kw OR 'maxill*':ti,ab,kw OR 'mandib*':ti,ab,kw OR 'jaw':ti,ab,kw) AND ('cancer':ti,ab,kw OR 'cancers':ti,ab,kw OR 'carcinoma':ti,ab,kw OR 'carcinomas':ti,ab,kw OR 'tumor':ti,ab,kw OR 'tumour':ti,ab,kw OR 'tumors':ti,ab,kw OR 'tumours':ti,ab,kw OR 'malignan*':ti,ab,kw OR 'squamous cell carcinoma*':ti,ab,kw) OR 'oral cancer':ti,ab,kw OR 'oscc':ti,ab,kw OR 'oral squamous cell carcinoma':ti,ab,kw OR 'hnscc':ti,ab,kw OR 'oral carcinoma':ti,ab,kw OR 'oral cancers':ti,ab,kw OR 'oral carcinomas':ti,ab,kw OR 'oral malignan*':ti,ab,kw OR 'oral tumors':ti,ab,kw OR 'oral tumours':ti,ab,kw OR 'oral tumour':ti,ab,kw OR 'oral tumor*':ti,ab,kw OR 'mouth cancer'/exp OR 'tongue cancer'/exp) AND ('border*':ti,ab,kw OR 'margin*':ti,ab,kw OR 'tumor free resection*':ti,ab,kw OR 'surgical margin'/exp OR 'thickness'/exp OR 'depth of invasion'/exp) AND [2010-2023]/py.
